# Editorial: Crop resistance mechanisms to alleviate climate change-related stress

**DOI:** 10.3389/fpls.2024.1368573

**Published:** 2024-02-05

**Authors:** José Ramón Acosta-Motos, Juan D. Franco-Navarro, María José Gómez-Bellot, Sara Álvarez

**Affiliations:** ^1^ Plant Biotechnology for Food and Agriculture Group (BioVegA), Universidad Católica San Antonio de Murcia (UCAM), Murcia, Spain; ^2^ Plant Biotechnology, Agriculture and Climate Resilience Group, Associate Unit of R&D+i CSIC-UCAM, Murcia, Spain; ^3^ Plant Ion and Water Regulation Group, Instituto de Recursos Naturales y Agrobiología de Sevilla (IRNAS, CSIC), Seville, Spain; ^4^ Hygiene Quality and R&D Department, CLECE S.A., University Hospital of Puerto Real (HUPR), Cádiz, Spain; ^5^ Irrigation Department, Centro de Edafología y Biología Aplicada del Segura (CEBAS-CSIC), Murcia, Spain; ^6^ Unit of Woody and Horticultural Crops, Instituto Tecnológico Agrario de Castilla y León (ITACyL), Valladolid, Spain

**Keywords:** climatic change, environmental shifts, global warming, plant breeding, plant resilience, sustainable agriculture

Anthropogenic activities have aggravated the effects of global climate change on ecosystems (IPCC, 2018). Plants are sessile organisms unable to escape from an adverse environment and for this reason they suffer to a great extent from stresses, which can negatively impact their growth and development (Aroca, 2012; Gull et al., 2019). Global warming is increasingly causing extreme climatic situations such as very high or low temperatures, drought and flooding events, hailstorms, wildfires, extreme precipitation events, and the reduction of fertile soil through desertification and salinization (Comas et al., 2013; FAO, 2016; Acosta-Motos et al., 2017; Franco-Navarro et al., 2021). In addition to this, warmer temperatures and higher humidity related to climate change can also increase pest and disease pressure on plants by altering the geographic range, population size, and timing of pest and disease outbreaks. Taken together abiotic stress related with climate change, such as drought or extreme temperature, may exacerbate the spread and severity of various diseases associated with biotic stress, increasing the vulnerability of plants to pathogens (some examples include insects, fungi, bacteria or viruses) (IPPC Secretariat, 2021).

Biotic and abiotic stresses affect plant growth and development. Since the development of a plant includes its vegetative and reproductive development, any stress that affects these processes is important to consider as it could cause irreversible damage to the plant and could ultimately lead to its death (Gull et al., 2019). The most relevant issue of climate change’s related-stresses in plants is the decrease in crop production (Lobell & Gourdji, 2012). Considering that the growing world population is predicted to reach 9.7 billion in 2050, global efforts are being made to increase food resources, improving crop or agronomic practices, especially in developing countries (Tilman et al., 2002; Godfray et al., 2010; Jurado et al., 2024). However, there are other issues promoted by climate change, affecting native and ornamental plants (Álvarez et al., 2019), floricultural production (Kumar et al., 2013), among others.

This editorial introduces a Research Topic which collects publications that study in detail the resilience mechanisms (tolerance and/or resistance) developed by plants to successfully cope with different biotic and abiotic stresses related to climate change at morphological, physiological, biochemical, and molecular levels (**Figure 1**). By promoting discussions on innovative farming practices for environmental mitigation and sustainable food production, it aims to provide a guide for resilient ecosystems and empower researchers, farmers and policymakers to manage climate change challenges.

**Figure 1:**
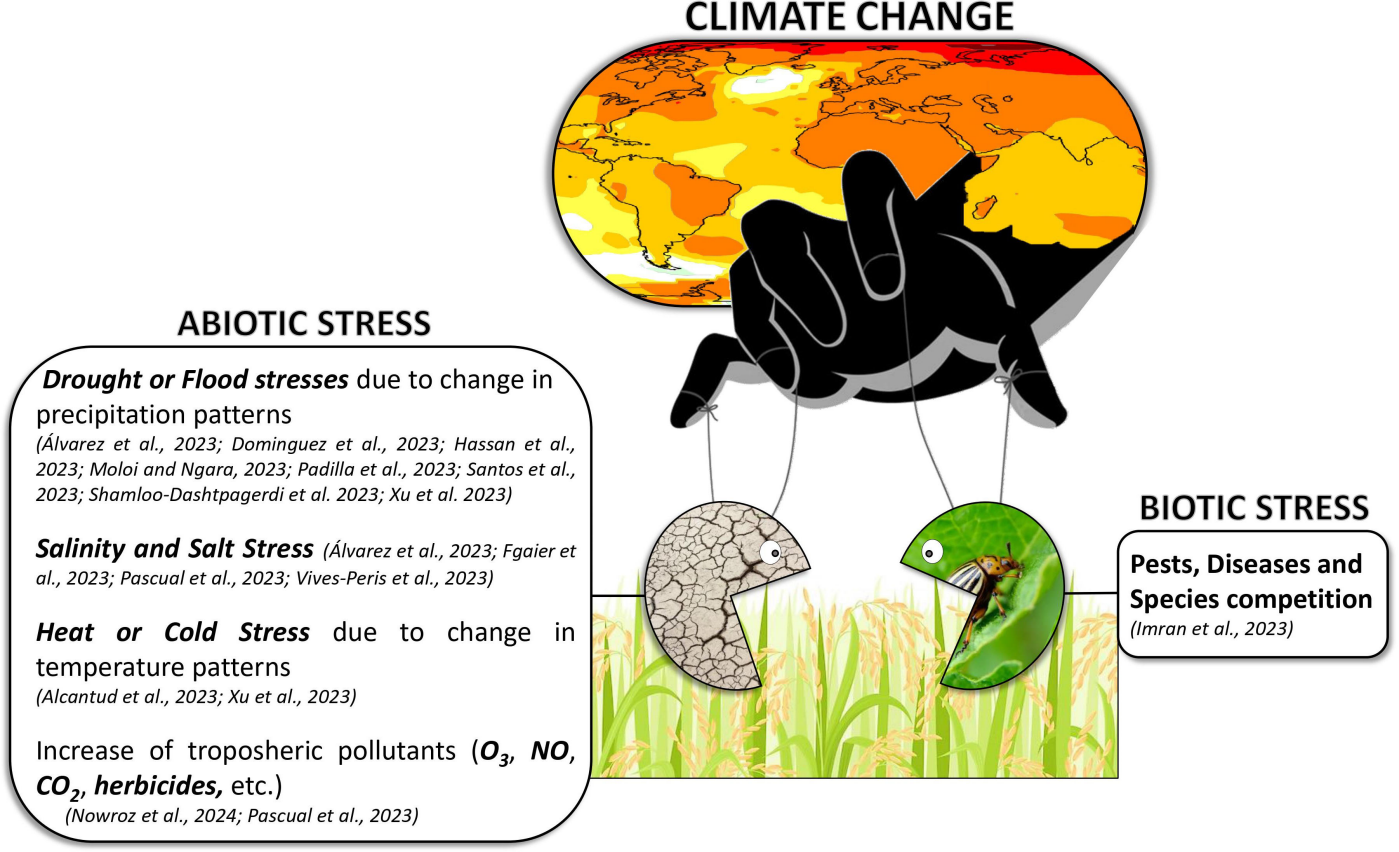
This figure represents a brief outline/summary of the Research Topic including its references.

Each publication compiled in this Research Topic is focused on a specific sort of climate change’s related-stress (or a combination of two, i.e., drought and salinity), and all of them are presented below.

Drought or water scarcity is one of the major constraints limiting crop production worldwide. Water deficit applied to different plant species of the family Gramineae (*Trichloris crinita*, Dominguez et al. *; Rice - Oryza* sp., Hassan et al. *; Wheat - Triticum aestivum*, Shamloo-Dashtpagerdi et al. *), or* Solanaceae *(Capsicum annuum*, Padilla et al. ), revealed significant variation in morphological, physiological, biochemical, and molecular levels. Tolerant variants and specific markers were screened supporting their integration into breeding programs to develop new plant varieties tolerant to drought stress (*Trichloris crinita*, Dominguez et al.; and Rice, Hassan et al.). Besides, Hassan et al. underscores the role of Molecular Assisted Selection (MAS) in advancing varietal development, emphasising the need for further exploration of genes and Quantitative Trait Loci (QTLs). Other works study the effect of changes in phytohormone modulation to enhance drought tolerance in pepper plants (Padilla et al., 2023); reveal a drought-responsive miRNA-target modules (miR1119-MYC2), which emerges as a potential biomarker for assessing wheat genotypes’ drought tolerance levels (Shamloo-Dashtpagerdi et al. ); or review the critical role of protein degradation as a marker for the selection of drought-tolerant genotypes (Moloi and Ngara).

Water deficit and salt stress were explored in the Mediterranean Anacardiaceae *Pistacia lentiscus* by Álvarez et al.  employing moderate and severe deficit irrigation, along with using saline water (around 4 dS m^-1^ salinity). Under these conditions, *Pistacia* exhibits favourable behaviour, making it suitable for landscaping in arid and saline regions.

A review about the effect of water deficit in combination with heat in plants highlights the changes of protein markers in both treatments, and emphasises the decrease of protein abundance, and its structure and stability, in addition to changes in the expression pattern of post-translational modifications (PTMs) and differentially expressed proteins (DEPs), which are also available markers for plant breeding (Xu et al*.* ).

Salinity and salt stress is one of the most important abiotic stresses. Vives-Peris et al. focused on the selection of morphological, physiological, and molecular markers to form the basis for future breeding programs, guiding the selection of optimal rootstock-scion combinations tailored to specific conditions.

Fgaier et al. assayed salinity combined with seed *priming* effect (seeds exposed to UV-C light) as a mitigation mechanism for the effects of salt stress on Lettuce seeds (*Lactuca sativa*). Increased concentrations of Salicylic acid (SA) and cytokinins are likely to contribute to positive effects under high salinity.

The most important soil-associated abiotic stresses is due to the excessive accumulation of herbicides along with the increase of salinity in soils. Pascual et al. observed in tomato plants subjected to salt-stress conditions and in the presence of the herbicide Paraquat, a reduction in the oxidative damage, promoted by the protective effect of the exogenous application of spermine.

Among the abiotic environmental factors, abrupt change in temperature (heat and cold stress) is the most important factor which significantly affects life processes of all organisms. Alcantud et al. assayed, for 2 years, with the Veronicaceae *Antirrhinum majus* under three different temperature regimes: control (22/16°C), cold (15/5°C), and hot (30/23°C), suggesting that short-term heat or cold exposure induces changes in plant gene expression, affecting crucial plant life processes (seed production, flower development, flower colour, etc.). It emphasises ecological and economic implications of temperature-induced changes in floriculture.

Anthropogenic activities of the last century have changed the concentration of pollutants in the Troposphere (NO, CO_2_, O_3_, etc.). All those contaminant gases are in contact with animals, plants, soil, etc. Nowroz et al*.* (2024) gives light to the knowledge on plant defensive responses against contamination of tropospheric Ozone (O_3_), which levels are increasing mainly due to human activities. A reduction in the stomatal conductance and in the carbon fixation are the first symptoms of the interaction with O_3_, reducing the net photosynthetic rate and plant growth.

Another of the great challenges that crops face is biotic stress. Imran et al. highlight Trichoderma culture filtrates’ potent antifungal effect on *Alternalia solani* mycelial growth, demonstrating strong inhibitory potential. In greenhouses and fields, these filtrates not only decrease early blight infection, but also promote plant growth and fruit production, serving as effective plant growth promoters. This practice contributes to sustainable agricultural production by mitigating the risk of fungicide-resistant early blight pathogens.

Finally, Santos et al. review the recurrent pressing issue of fruit cracking and highlights its potential for molecular breeding research, driven by genetic factors. Omics technologies offer molecular-level insights into this disorder. While direct evidence through mutations is lacking, the study identifies exocarp-specific transcripts crucial for cracking development, involving cuticular membrane, cell wall mechanisms, and wax biosynthesis. With climate change on the horizon, understanding plant responses at the transcriptomic level is deemed crucial for effective molecular breeding and enhancing crop resilience

This Research Topic delves into the intricate challenges posed by global warming and environmental shifts. Acknowledging the contributions of each article, we extend our gratitude to the authors for their valuable insights. Their exploration of diverse plant responses, spanning molecular mechanisms, breeding strategies, and ecological implications, paves the way for sustainable agriculture. Special thanks to the dedicated reviewers for ensuring the quality and depth of these contributions.

## Author contributions

JA-M: Writing – original draft, Writing – review & editing. JF-N: Writing – original draft, Writing – review & editing. MG-B: Writing – original draft, Writing – review & editing. SÁ: Writing – original draft, Writing – review & editing.

## References

[B1] Acosta-Motos J. R. Ortuño M. F. Bernal-Vicente A. Diaz-Vivancos P. Sanchez-Blanco M. J. Hernandez J. A. (2017). Plant responses to salt stress: Adaptive mechanisms. Agronomy 7 (1), 18. doi: 10.3390/agronomy7010018

[B2] Álvarez S. Gómez-Bellot M. J. Acosta-Motos J. R. Sánchez-Blanco M. J. (2019). Application of deficit irrigation in *Phillyrea angustifolia* for landscaping purposes. Agric. Water Manage. 218, 193–202. doi: 10.1016/j.agwat.2019.03.049

[B3] Aroca R. (2012). Plant responses to drought stress (Berlin/Heidelberg, Germany: From Morphological to Molecular Features; Springer), 1–5.

[B4] Comas L. H. Becker S. R. Cruz V. M. Byrne P. F. Dierig D. A. (2013). Root traits contributing to plant productivity under drought. Front. Plant Sci. 4. doi: 10.3389/fpls.2013.00442 PMC3817922 24204374

[B5] FAO (2016). The state of food and agriculture. Climate change, agriculture and food security (Rome: Food and Agriculture Organization of the United Nations (FAO). Available at: http://www.fao.org/3/a-i6030e.pdf.

[B6] Franco-Navarro J. D. Díaz-Rueda P. Rivero-Núñez C. M. Brumós J. Rubio-Casal A. E. de Cires A.. (2021). Chloride nutrition improves drought resistance by enhancing water deficit avoidance and tolerance mechanisms. J. Exp. Bot. 72 (14), 5246–5261. doi: 10.1093/jxb/erab143 33783493 PMC8272566

[B7] Godfray H. C. J. Beddington J. R. Crute I. R. Haddad L. Lawrence D. Muir J. F.. (2010). Food security: the challenge of feeding 9 billion people. Science 327, 812–818. doi: 10.1126/science.1185383 20110467

[B8] Gull A. Lone A. A. Wani N. U. I. (2019). Biotic and abiotic stresses in plants.Abiotic and biotic stress in plants, 1–19. Ed. IntechOpen Limited, London, UK, ISBN: 978-1-83962-485-8. doi: 10.5772/intechopen.77845

[B9] IPCC (2018). Global warming of 1.5 °C: An IPCC special report on the impacts of global warming of 1.5 °C above pre-industrial levels and related global greenhouse gas emission pathways, in the context of strengthening the global response to the threat of climate change, sustainable development, and efforts to eradicate poverty. Eds. Masson-Delmotte V. Zhai P. Pörtner H.-O. Roberts D. Skea J. Shukla P. R. Pirani A. (Geneva, Switzerland: IPCC). Available at: https://www.ipcc.ch/site/assets/uploads/sites/2/2019/06/SR15_Full_Report_High_Res.pdf. 630 pp.

[B10] IPPC Secretariat (2021). Scientific review of the impact of climate change on plant pests – A global challenge to prevent and mitigate plant pest risks in agriculture, forestry and ecosystems (Rome: FAO on behalf of the IPPC Secretariat). doi: 10.4060/cb4769en

[B11] Jurado C. Díaz-Vivancos P. Gregorio B. E. Acosta-Motos J. R. Hernández J. A. (2024). Effect of halophyte-based management in physiological and biochemical responses of tomato plants under moderately saline greenhouse conditions. Plant Physiol. Biochem. 206, 108228. doi: 10.1016/j.plaphy.2023.108228 38043255

[B12] Kumar P. N. Kadam G. B. Kumar R. (2013). “Floriculture, a viable option of diversification in the light of climate change,” in Climate-Resilient Horticulture: Adaptation and Mitigation Strategies (India: Springer India), 213–220. doi: 10.1007/978-81-322-0974-4_19

[B13] Lobell D. B. Gourdji S. M. (2012). The influence of climate change on global crop productivity. Plant Physiol. 160 (4), 1686–1697. doi: 10.1104/pp.112.208298 23054565 PMC3510102

[B14] Tilman D. Cassman K. G. Matson P. A. Naylor R. Polasky S. (2002). Agricultural sustainability and intensive production practices. Nature 418, 671–677. doi: 10.1038/nature01014 12167873

